# IrCytoToxDB: a dataset of iridium(III) complexes cytotoxicities against various cell lines

**DOI:** 10.1038/s41597-024-03735-w

**Published:** 2024-08-10

**Authors:** Lev Krasnov, Sergei Tatarin, Daniil Smirnov, Stanislav Bezzubov

**Affiliations:** grid.4886.2 0000 0001 2192 9124 N.S. Kurnakov Institute of General and Inorganic Chemistry, Russian Academy of Sciences, Leninskii pr. 31, Moscow, 119991 Russia

**Keywords:** Ligands, Cancer, Cheminformatics

## Abstract

Iridium(III) complexes nowadays became rising stars in various health-related applications. Thus, there is a necessity to assess cytotoxicity of the synthesized molecules against cancer/normal cell lines. In this report, we present a dataset of 2694 experimental cytotoxicity values of 803 iridium complexes against 127 different cell lines. We specify the experimental conditions and provide representation of the complexes molecules in machine-readable format. The dataset provides a starting point for exploration of new iridium-based cellular probes and opens new possibilities for predictions of toxicities and data-driven generation of new organometallic anticancer agents.

## Background & Summary

Over the past decades, cyclometalated iridium (III) complexes have garnered significant attention, primarily because of their unique physicochemical properties (bright luminescence, thermodynamic and kinetic stability, ability to effectively generate reactive oxygen species) and the wide tuneability of such properties accessed by ligands variation in the metal coordination sphere^1^. Abovementioned properties determine their popularity as emitters in phosphorescent light-emitting diodes^2–4^, as photocatalysts in various organic transformations^5,6^ and in hydrogen evolution reactions^7,8^ and as photosensitizers in solar cells^9,10^. Moreover, they have recently emerged as great performers in biological applications^11–13^. These vary from different types of imaging – staining^14,15^, bioimaging^16,17^, biosensing^18,19^ tasks to chemotherapy/photodynamic therapy agents^20–22^. The other popular class of the iridium complexes is based on the cyclopentadiene anion (Cp^−^) framework, being also explored as chemotherapeutic agents^23,24^. Such roles have opposite requirements to the cytotoxicity values – complexes capable of biosensing should possess the lowest toxicity possible, whereas complexes accused of therapeutic roles need to demonstrate moderate-to-high cytotoxicity with the highest selectivity possible towards the cancer cell lines. For photodynamic therapy purposes the corresponding complexes should demonstrate high selectivity indexes (low cytotoxicity in the dark with high cytotoxicity under irradiation)^25^. It can be unambiguously seen that for all these applications evaluation of cytoxicity is a critical aspect which guides the molecular design.

In order to develop new cyclometalated iridium(III) complexes that can effectively serve as biosensors/medicines, it is necessary to understand their structure-property relationships, at least in terms of cytotoxicity. However, due to the structural diversity of these compounds (i.e. variability of two types of ligands), revealing such relationships just from the chemical point of view is not straightforward. In this regard, prediction of target properties as well as data-driven exploration of new molecules with desired properties is extremely helpful, providing significant acceleration of the molecular design. Nowadays machine-learning methods emerged as effective way to estimate cytotoxicity of various organic compounds^26–28^. For this purpose, curating literature-based datasets might be the suitable way to obtain desired data to train models without performing extensive synthetic and biochemical work^29^. Moreover, recently the data-driven approach was extended to organometallic compounds, probing the antibacterial activity of half-sandwich ruthenium complexes and predicting novel molecules of this type^30^.

However, the cytotoxicity data for iridium(III) complexes is quite scattered among dozens of sources. An additional complexity is created by huge variety of experimental conditions of cytotoxicity evaluation, what hinders direct comparison of results obtained by different scientific groups. Herein we present a dataset of experimental cytotoxicity values for iridium(III) complexes reported in the 222 literature papers from 2008 to 2022. The dataset contains 2694 values of cytotoxicities with specification of the experimental parameters and the cell lines. The chemical space could be mainly divided in two parts – the bis-cyclometalated (containing two cyclometalated ligands) iridium(III) complexes and half-sandwich iridium(III) complexes, with the formula being presented in machine-readable format (SMILES^31^). To the best of our knowledge, this is the first data survey for transition metal compounds cytotoxicities. These data can assist in guiding the synthesis of novel complexes for biological applications, in screening for structures or substructures of interest, in probing cytotoxicity values, or in training machine learning and deep learning models for various tasks.

## Methods

Firstly, the list of relevant peer-reviewed journals was selected. In particular, the most high-impact journals from Springer Nature, American Chemical Society, Royal Society of Chemistry, Wiley, MDPI and Elsevier publishers considering topics in: inorganic and organometallic chemistry, medicinal chemistry and general chemical sciences were chosen. The motivation behind choosing such journals was that they systematically publish articles considering either iridium(III) complexes or cytotoxicity studies.

Secondly, for the total of 36 journals articles containing fragment “iridium” or “Ir(III)” in the title were extracted using freely available Cobalt search engine (an example of search query is as follows: https://cobalt.colab.ws/?term=ir(III)&publisher_id=17&journal_id=6830&year_to=2022 and https://cobalt.colab.ws/?term=iridium&publisher_id=17&journal_id=6830&year_to=2022). The resulting 6247 articles were manually screened for cytotoxicity data. Given that the Cobalt search engine performs only within article titles, use of specific keywords narrows the output significantly. For example, the most general query “iridium” for the articles published until 2023 returns 17450 results (https://cobalt.colab.ws/?term=iridium&year_to=2022), whereas the query “iridium anticancer” returns 136 results (https://cobalt.colab.ws/?term=iridium%20anticancer&year_to=2022), “iridium cytotoxicity” (https://cobalt.colab.ws/?term=iridium%20cytotoxicity&year_to=2022) returns only 22 results and the query “iridium toxicity” returns only 6 results (https://cobalt.colab.ws/?term=iridium%20toxicity&year_to=2022). In our work we aimed at maximizing the dataset size within the array of journals specified in the article. Thus, manual filtering is the most reliable way to do it.

**Fig. 1:**
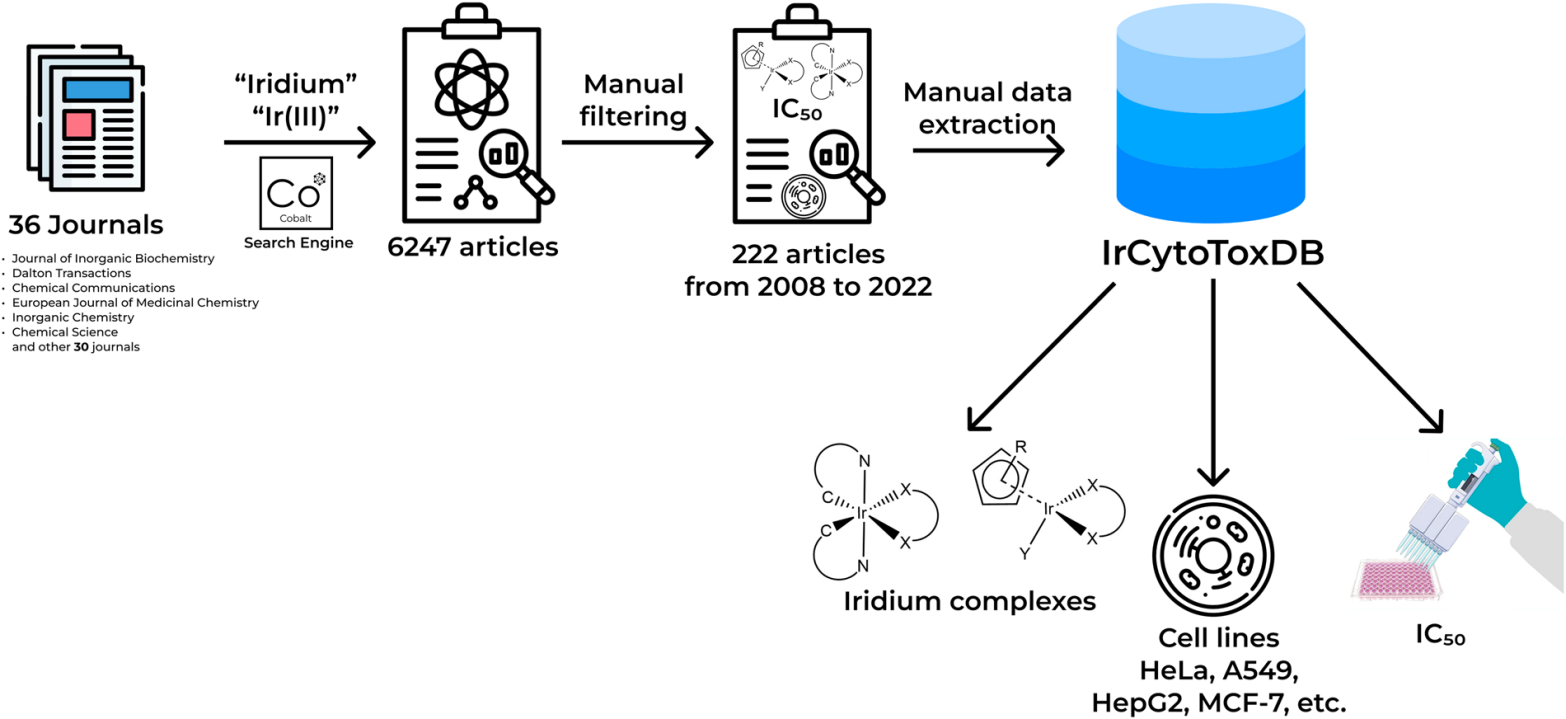
The algorithm of data collection.

As a result, 222 articles from 2008 to 2022 were chosen and the data was manually extracted into a CSV file. SMILES for ligands (L1, L2, L3, L4) were generated by ChemDraw 18.0 and were canonized using the open-source cheminformatics software RDKit (https://www.rdkit.org). The IC_50_ (a quantitative measure that indicates how much of a particular inhibitory substance (e.g. drug) is needed to inhibit, *in vitro*, a given biological process by 50 percent) was chosen as the most common metric to represent cytotoxicity *in vitro*^32^. The total list of journals as well as number of articles before and after manual filtering are presented in Table 1. The full algorithm of IrCytoToxDB formation is summarized in Fig. 1.

**Table 1:** The list of selected journals and number of articles before and after screening.

Journal	Total articles	Relevant articles
Journal of Inorganic Biochemistry	46	26
Dalton Transactions	556	25
Chemical Communications	390	18
European Journal of Medicinal Chemistry	21	14
Inorganic Chemistry	913	14
Chemical Science	101	12
Journal of Biological Inorganic Chemistry	19	10
Angewandte Chemie - International Edition	225	9
Dyes and Pigments	127	8
Journal of Organometallic Chemistry	689	8
Inorganica Chimica Acta	326	6
Journal of Medicinal Chemistry	10	6
Inorganic Chemistry Frontiers	32	6
Organometallics	790	6
Inorganic Chemistry Communications	71	5
Chemistry—A European Journal	150	5
New Journal of Chemistry	108	5
ZAAC	76	4
Journal of the American Chemical Society	795	4
RSC Advances	122	4
Scientific Reports	35	3
Polyhedron	161	2
European Journal of Inorganic Chemistry	239	2
ACS Applied Materials & Interfaces	80	2
ChemBioChem	4	2
Biomaterials	17	2
ChemMedChem	5	2
Pharmaceutics	3	2
Chemico-Biological Interactions	4	2
Journal of Materials Chemistry B	22	2
Chemistry—An Asian Journal	55	1
ChemPlusChem	7	1
Applied Organometallic Chemistry	11	1
Molecules	33	1
ACS Medicinal Chemistry Letters	2	1
Bioorganic Chemistry	2	1

## Data Records

IrCytoToxDB can be accessed online within Zenodo^33^. The main dataset is structured as a downloadable CSV format data record. Description of each available metadata field is provided in Table 2. Speaking about the molecular representation of the complexes, additional remarks should be done.

**Table 2:** Description of each metadata field.

Column	Description	Type
L1	SMILES representation of the L1 ligand attached to the iridium ion	string
L2	SMILES representation of the L2 ligand attached to the iridium ion	string
L3	SMILES representation of the L3 ligand attached to the iridium ion	string
L4	SMILES representation of the L4 ligand attached to the iridium ion	string
Counterion	SMILES representation of the counterion (if the complex molecule is charged)	string
Abbreviation_in_the_article	the original abbreviation depicting the complex in the article	string
IC50Dark(M*10^−^6)	value of IC_50_ originally presented in the article	float or string
IC50Dark_standard_error(M*10^−^6)	standard error of IC_50_ originally presented in the article	float
IC50Light(M*10^−^6)	value of IC_5__0_ under irradiation originally presented in the article	float or string
IC50Light_standard_error(M*10^−^6)	standard error of IC_50_ under irradiation originally presented in the article	float
Excitation_Wavelength(nm)	excitation wavelength related to IC_50_ Light values	float or string
Irradiation_Time(minutes)	irradiation time related to IC_50_ Light values	float
Irradiation_Power(W*m^−^2)	power of light source related to IC_50_ Light values	float
Cell_line	cell line (HeLa, A549, etc.)	string
Time,h	time of exposure of the complexes to the cell line	float
DOI	doi of a data source for given values	string
Year	year of a data source for given values	float
Comments	additional comments	string

The array of iridium(III) complexes could be mainly divided in two parts – *bis*-cyclometalated Ir(III) complexes and half-sandwich Ir(III) complexes. The former usually contain two cyclometalated ligands and one or two ancillary (or third cyclometalated) ligand; for these L1 and L2 correspond to the cyclometalated ligands and L3 (or L3 and L4) corresponds to the ancillary ligand. The latter usually contain one cyclopentadiene^−^(Cp^−^)-based ligand, one bidentate ligand and one monodentate ligand; for these L1 corresponds to the Cp^−^-based ligand, L2 corresponds to the bidentate ligand and L3 corresponds to the monodentate ligand.

Some ligands make formally covalent bonds with the Ir(III) ion. For these a negatively charged bond-forming atom is drawn in the SMILES of corresponding ligand.

**Fig. 2:**
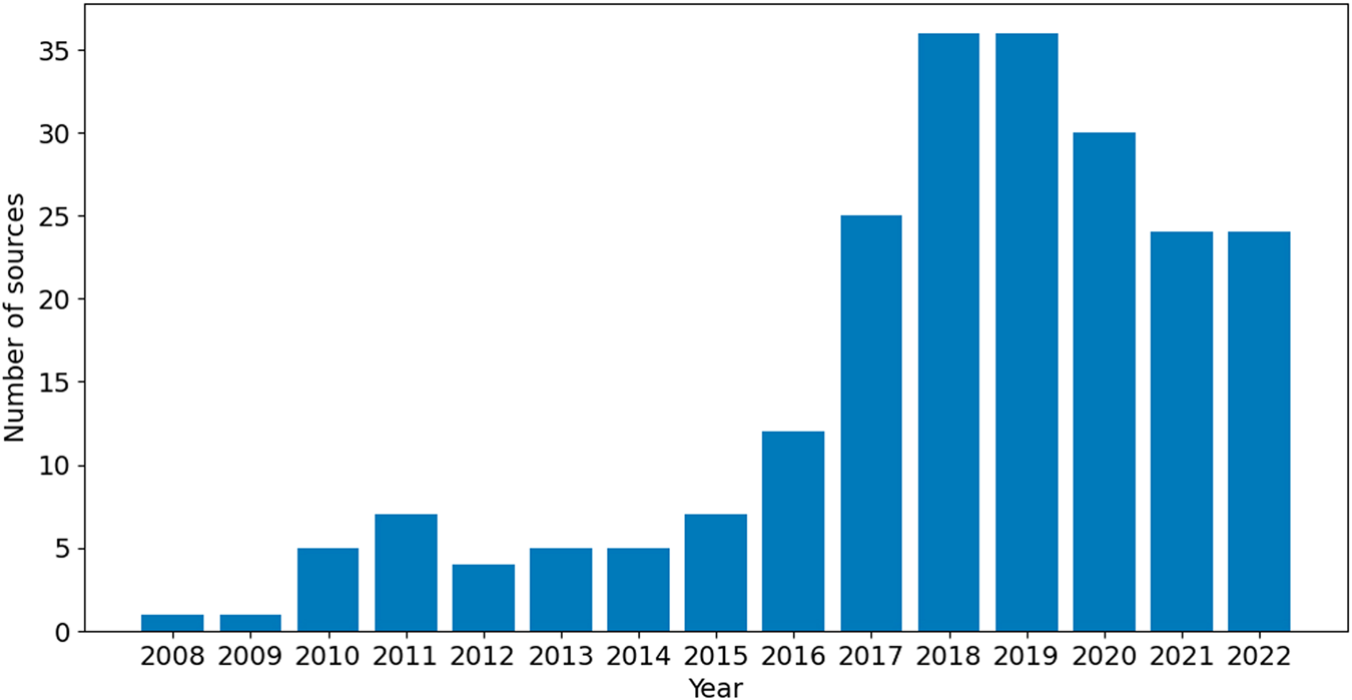
Distribution of the data sources by year.

**Fig. 3:**
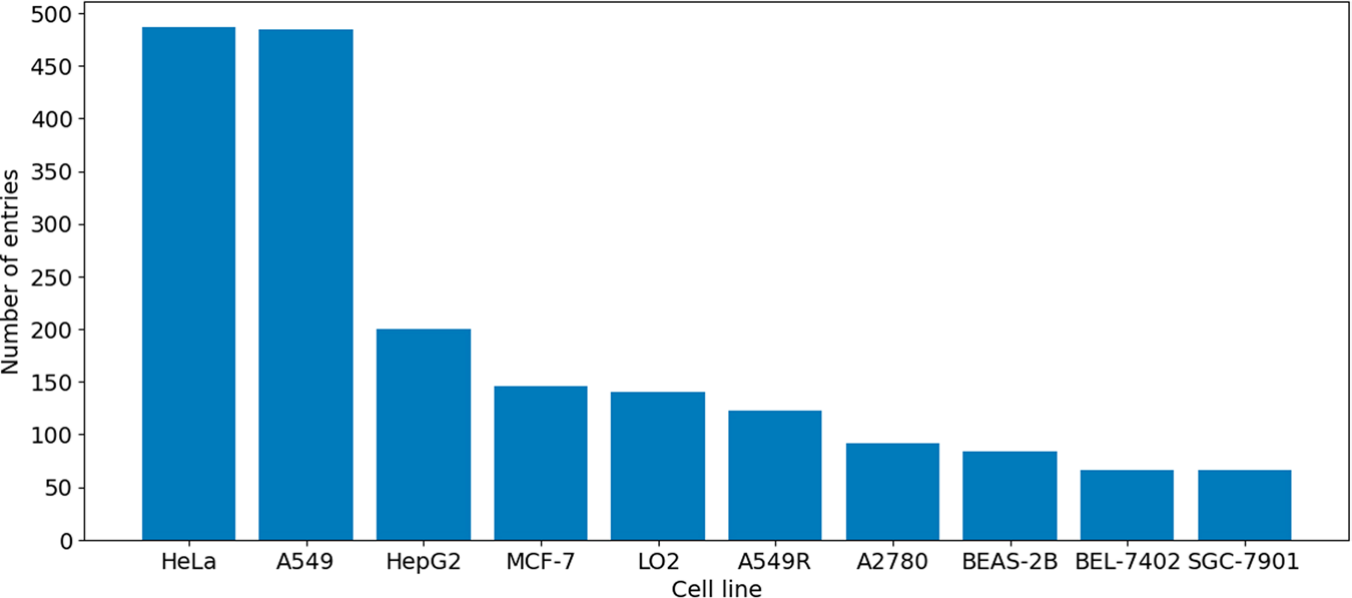
Distribution of the data by cell line (10 most popular are shown).

**Fig. 4:**
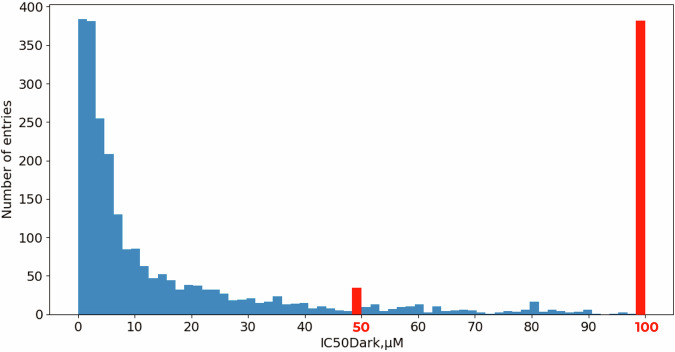
Distribution of the data by IC_50_ values for all the cell lines. The bars 50 *μ*M and 100 *μ*M are depicted in red because they represent additional values, which were presented in original articles as  >50 *μ*M and as  >100 *μ*M, respectively.

IrCytoToxDB contains 2694 experimentally measured cytotoxicity values of 803 unique iridium(III) complexes against 127 different cell lines reported in the 222 literature papers from 2008 to 2022. The distribution of the data amongst the publication year explicitly shows gradual increase of scientific interest to the Ir(III) complexes in biological applications (Fig. 2). Amongst the cell lines two almost equally popular stand out – HeLa and A549, with others having significantly less entries (Fig. 3). Nevertheless, 10 most popular cell lines make up 70 percent of the extracted data. Distribution of the toxicity data itself clearly depicts two main groups of compounds – possessing extremely high (<10 *μ*M) or extremely low (>100 *μ*M) cytotoxicity (Fig. 4), both representing the vast majority of explored iridium(III) complexes.

## Technical Validation

The presented experimental data has been already published in peer-reviewed scientific journals. Thus, potential errors in our dataset can be formally divided into two categories: missed articles and mistakes in data extraction.

### Missed articles

Missed articles refers to not incorporating relevant articles into the dataset. The search criteria needed to narrow the space of papers to those clearly dealing with iridium and its compounds. For this purpose, the search was performed in the titles of articles by the word “iridium” itself and the abbreviation “Ir(III)”. The resulting space of papers was broad, requiring removal of vast majority of unsuitable articles (dealing with iridium(III) complexes applied in optoelectronics/photonics or catalysis). It should be noted that Potentially missed articles may be published in some regional journals (commonly not in English language, that hampers their analysis and extraction of data). We have also performed primary article extraction using Google Scholar search engine (https://scholar.google.com/scholar?&as_sdt=0%2C5&q=iridium). However, their search algorithms are much more broad resulting in far more results (e.g. searching term “iridium” for the articles published until 2023 returns 302000 results), which are barely screenable, and cannot perform search within the specified journals that slows down data collection significantly. Still, this trial did not show any noticeable drawbacks in our self-made algorithm. Moreover, we understand that not all research data is contained in peer-reviewed articles (e.g., government reports, patents), however, these were outside the scope of the dataset in its current capacity.

### Mistakes in data extraction

The data extraction was performed with participation of specialists in coordination chemistry, who have vast experience in working with iridium(III) complexes. The data was subjected to cross-checking to ensure consistency and validity of the dataset. The cross-validation technique was adapted from^34^. Two people with sufficient experience in coordination chemistry and describing the complexes’ properties, separately collected the cytotoxicity data from the papers. The third person checked these two datasets and added them to the final dataset. The inconsistencies were double-checked, and the correct variant was added. After that the resulting dataset was subjected to a rechecking to ensure completeness and clarity of the data.

## Usage Notes

The objective of collecting and sharing these data can be divided in two parts: 1) To encourage research on new iridium(III) complexes for biological applications, and 2) to propose a benchmark dataset for accelerating the discovery of such molecules. In particular, one can evaluate chemically-biased structure-property correlations or perform meta-analysis of the data for target synthesis of selectively cytotoxic or non-cytotoxic iridium(III) complexes as therapeutics or bioimaging agents, respectively. Finally, the dataset paves the way to train machine learning and deep learning models for improved development of iridium(III) compounds for targeted health-related applications.

For further updates of the dataset we encourage researchers to use Cobalt search engine. However, it also should be noted that performing search in Google Scholar with additional keywords results in relatively specialized output (see e.g. https://scholar.google.com/scholar?as_ylo=2023&q=iridium+anticancer or https://scholar.google.com/scholar?as_ylo=2023&q=iridium+cytotoxicity), so it might also be considered as a reliable way to update the dataset. Still, as the Google Scholar cannot perform search within the specified journals, one needs to check the quality of sources, avoiding low-quality journals and preprint services. Finally, we have also tried Dimensions as an alternative search engine; using journals filtration with the “search in title and abstract” button gives a result similar to Cobalt for the query “iridium” (e.g. https://app.dimensions.ai/discover/publication?search_mode=content&search_text=iridium&search_type=kws&search_field=text_search&or_facet_source_title=jour.1358263). Thus, Dimensions can also be considered as a suitable search engine for updating the dataset.

## Data Availability

No custom code was used to collect and process the data described in this manuscript.
